# Governance and One Health: Exploring the Impact of Federalism and Bureaucracy on Zoonotic Disease Detection and Reporting

**DOI:** 10.3390/vetsci2020069

**Published:** 2015-05-13

**Authors:** Heather A. Allen

**Affiliations:** Center for Food Security and Public Health, College of Veterinary Medicine, Iowa State University, Ames, IA 50011, USA; E-Mail: hallen@iastate.edu; Tel.: +1-515-294-7189; Fax: +1-515-294-8259

**Keywords:** federalism, One Health, public health, animal health, disease reporting, disease detection, zoonoses, institutions, governance

## Abstract

The merits of One Health have been thoroughly described in the literature, but how One Health operates in the United States federal system of government is rarely discussed or analyzed. Through a comparative case-study approach, this research explores how federalism, bureaucratic behavior, and institutional design in the United States may influence zoonotic disease outbreak detection and reporting, a key One Health activity. Using theoretical and empirical literature, as well as a survey/interview instrument for individuals directly involved in a past zoonotic disease outbreak, the impacts of governance are discussed. As predicted in the theoretical literature, empirical findings suggest that federalism, institutional design, and bureaucracy may play a role in facilitating or impeding zoonotic disease outbreak detection and reporting. Regulatory differences across states as well as compartmentalization of information within agencies may impede disease detection. However, the impact may not always be negative: bureaucracies can also be adaptive; federalism allows states important opportunities for innovation. While acknowledging there are many other factors that also matter in zoonotic disease detection and reporting, this research is one of the first attempts to raise awareness in the literature and stimulate discussion on the intersection of governance and One Health.

## 1. Introduction

Beginning with Calvin Schwabe, the merits of a One Health approach have been thoroughly described for over three decades [1,2]. In the United States, the government has the duty, obligation, and authority to protect environmental health, public health, as well as animal health [3,4]. However, little has been written on how the U.S. system of governance impacts One Health. This research uses zoonotic disease detection and reporting in the United States to examine to what extent federalism, bureaucratic behavior, and design of U.S. institutions may impact this key set of One Health activities.

Zoonotic disease detection and reporting is critical to One Health; over half of all pathogens known to infect humans are zoonotic and an estimated 75 percent of emerging infectious diseases are zoonotic [5]. Yet, animal and human disease surveillance, detection, and reporting activities in the United States are fragmented, both practically and politically. There are disconnects between the animal health and public health sectors, as well as a myriad of state and federal surveillance systems [6]. The National Strategy for Countering Biological Threats specifically called for improving “coordination between human, plant, and veterinary disease reporting systems” [7] (p. 6) and “fostering information sharing” [7] (p. 12) between different government sectors. A delay in the detection and reporting of a zoonotic disease outbreak can have significant implications for public health and animal health [8]. While quantitative research has been conducted to analyze timeliness in disease detection and reporting [9,10], this research uses a qualitative approach to frame One Health in the context of the U.S. system of governance, exploring the complexities of zoonotic disease detection in the United States.

To start, it is necessary to describe the U.S. system of governance, including theories of bureaucratic behavior and federalism, focusing on potential impacts to zoonotic disease detection. Next, a comparative case study provides further information based on data collected from publicly available literature and a survey/interview instrument. Four zoonotic disease outbreaks from 2000–2006 are selected for further analysis, based on the speed in which they were detected. Then, the factors impeding or facilitating detection are carefully reviewed to describe how or to what extent federalism, institutional design, and bureaucratic behavior may facilitate or impede rapid zoonotic disease detection and reporting. Ultimately, this research (1) provides information to raise awareness regarding the complexity of disease detection and reporting in a federal system and (2) stimulates further discussion and future research on the significant gap in the literature at the intersection of One Health and public policy and administration.

## 2. U.S. System of Governance & One Health

Many theories regarding both federalism and bureaucracy have been written; this section merely summarizes key points with direct application to zoonotic disease detection and reporting and ultimately One Health.

### 2.1. Definitions

For the purpose of this paper, zoonotic disease detection and reporting are discussed together, acknowledging they are independent terms/activities, both distinctly important to disease surveillance and response. Detection typically refers to the initial recognition of the disease or a disease outbreak [11]; reporting refers to an individual—regardless of position—reporting this case or outbreak to a regulatory authority at the local, state, or federal level. In theory, if a disease is detected it should be reported (per applicable state and federal regulation), though this is not always the case [12]. Indeed, the detector of the disease and the reporter may be the same entity: for example, both public health and animal health laboratories may detect an outbreak and message these results to a government agency. These two connected but separate activities are discussed in tandem here, as response to an outbreak relies on both detection and reporting.

### 2.2. Federalism

Federalism is the division or separation of powers between the U.S. federal government and state governments. It is specifically granted in the 10th Amendment to the U.S. Constitution: “The powers not delegated to the United States by the Constitution, nor prohibited by it to the States, are reserved to the States respectively, or to the people” [13]. Federalism was a critical reaction by the framers of the U.S. Constitution to the strong central power in England [14].

On the federal side, “federal public health powers flow from the U.S. constitution” [3] (p. 79). Both the Animal Health Protection Act (7 U.S.C. 8301 *et seq*.) and Public Health Services Act (42 U.S.C. 201) broadly define the authority for the federal government to act on matters of disease and disease transmission in animals and people, respectively. However, these powers are also limited by the 10th Amendment. While there are definitely federal actions related to zoonotic disease detection and reporting, historically the authority over these activities falls to the states.

In particular, states are reserved what are known as police powers [3]. It is these police powers which enable state governments to legally conduct public health infectious disease surveillance within a population of individuals [15]. It is also through these powers that states can and do mandate disease reporting within the state from laboratories, physicians, and sometimes other entities or individuals [3,16]. Typically, disease reporting in states occurs in two executive agency pipelines—one for animal diseases to animal health/agriculture departments, and one for human diseases to public health/human services departments. While state to federal disease reporting is generally voluntary (from a purely regulatory perspective), federally accredited veterinarians and others do have the obligation to report specific animal diseases to the federal government [16].

While discussing the merits of different political science theories could fill a book, it is useful to note several positions. Some argue we are in an era of competitive federalism, where states and the federal government compete over various domestic policy areas [17]. In general, the enumerated powers of the federal government have been widely interpreted by the Supreme Court [3]. Moreover, it is possible that federal authority could be much more expansive on issues of disease surveillance and reporting if there was the desire (or perhaps necessity) to act [18]. However, the federal government has historically been the provider of mere performance standards to encourage consistency across state disease surveillance and reporting activities.

### 2.3. Bureaucratic Behavior & Institutional Design

Similarly, there is extensive literature on the role of institutional design in policy-making and intergovernmental relations, for example [19,20]. Because zoonotic disease detection and reporting is largely an executive-branch activity, the most important aspect is the behavior of these executive agencies. At both the state and the federal level, policy responsibilities are dispersed amongst executive agencies, sometimes with functions that broadly overlap. For example, different agencies may each have infectious disease surveillance systems for various populations or diseases. Human health and animal health issues are delegated to separate agencies. Often, wildlife disease issues are in yet another agency/entity. At the federal level in the United States, just to provide an example of the many agencies involved in zoonotic disease detection, the U.S. Department of Health and Human Services, U.S. Department of Agriculture, U.S. Department of Homeland Security, U.S. Department of Defense, and U.S. Department of the Interior all play some role in the surveillance and detection of zoonotic diseases (and this list is certainly not all inclusive) [21].

However, executive agencies are not designed—nor do they function—equally: some receive significantly more support (financial and/or political) than others, based on their area of authority and jurisdiction. Indeed, human health agencies at the state and federal level are typically significantly better funded than agencies devoted to the policy areas of agriculture or wildlife [22,23]. Additionally, some executive agencies are significantly more influenced by the stakeholders they serve than are others. A popular hypothesis of bureaucratic behavior is that agencies constantly seek greater autonomy and authority *vis-à-vis* other agencies [24,25]. This type of behavior can lead to a lack of cooperation, or at the least, little enthusiasm for shared tasks between agencies, even with regard for a common goal. Practically, this may result in a critical lack of information sharing or communication between executive agencies, at both a state and a federal level. However, there are also theories which suggest that this battle for power is not fully explanatory: agencies act in specific ways for many different reasons, including the organizational culture, resources at hand, leadership, and reputation. Regardless of which theory one might subscribe to, it is clear that bureaucratic behavior that results from the design of our institutions may foster impediments to effective coordination and communication.

### 2.4. Potential Implications of Federalism, Bureaucratic Behavior, and Institutional Design for One Health

With regard to federalism, there are two primary issues which arise as concerns for the practice of zoonotic disease detection and reporting. First, because these powers are reserved for the states, the federal government does not have direct power—or has not typically exercised direct power—over how they are exercised. Second, when powers are reserved for the states, each state can, and does, choose to exercise these powers in various manners. Indeed, these issues are plainly observed by the widely varying requirements and capabilities for zoonotic disease detection and reporting in each state [12,16].

Bureaucracy and institutional design also may have important implications for One Health, specifically zoonotic disease detection and reporting. In particular, executive agencies are designed to often have overlapping responsibilities. Furthermore, they are often poor coordinators and cooperators. Due to the competing nature of executive agencies, for funding, influence, or authority, knowledge about specific disease cases can be compartmentalized, rather than shared between practitioners or officials at various levels; state or federal executive agencies may not cooperate or coordinate effectively on detection and reporting efforts [26].

Now that the stage has been set regarding how federalism and bureaucratic behavior may influence zoonotic disease detection and reporting, the research that follows works to unravel whether these potential implications are supported by empirical evidence.

## 3. Methods

A qualitative case study approach is employed, using a comparative case study as described in [27]. This research was accepted as exempt by The George Washington University Institutional Review Board, #0011107. A case study approach is not appropriate to examine “the prevalence of phenomena”; it is intended to provide a detailed description of how and why [27] (p. 10). This method uses a set of individual outbreaks to explore any impact of the U.S. system of governance on zoonotic disease detection and reporting.

### 3.1. Cases

The unit of analysis—or case—is a zoonotic disease outbreak. From prior research [28], the most rapidly detected outbreaks and the least rapidly detected outbreaks are identified. More than one type of each case is preferred [27], so two cases are identified from each group. In addition to being an outlier, each case also required existing, published literature regarding its detection, reporting, and/or response. With these criteria, the slowest outliers (least rapidly detected) are two salmonella outbreaks, one in 2006–2007 and one in 2003–2004. The two fastest outliers (most rapidly detected) are a case of rabies in 2006, and a case of anthrax in 2000. For simplicity, these are sometimes referred to as “slow” and “fast” outbreaks or cases, with the clear acknowledgment this is time to detection not speed of disease spread.

### 3.2. Data Sources

There are three key sources of information for this case study: publicly available literature specifically on the cases, the survey instrument/interviews, and theories of governance (discussed above). For each case, an open-source literature review was conducted, followed by a structured interview/survey for additional information. The information and evidence from these data sources were integrated, subsequently embedding qualitative results in a theoretical context.

### 3.3. Interview/Survey

Subjects were contacted by email and provided details on the research and an informed consent form. The subjects were identified from published articles and professional references (with express permission of the individual being contacted), and included practitioners, state officials, diagnostic laboratory personnel, and federal officials. Both animal health and public health subjects participated. Initially all potential respondents were involved in the fast and slow outlier cases. However, to improve sample size, the population was expanded to those who had specific experience responding to one of the zoonotic disease outbreaks identified in [28]. These participants were referred by other respondents.

Both structured interviews and a survey instrument were employed. The questions were the same whether the interview was conducted orally or the respondent replied to an online survey. This option was provided to offer individuals flexibility and improve the response rate. Respondents were asked about the factors they thought improved or impeded zoonotic disease outbreak detection and reporting in the specific zoonotic disease outbreak (case). They were also asked about the interaction between the animal health and human health sector and explicitly about whether federalism and institutional design impacted zoonotic disease detection and reporting. All questions were open-ended.

All responses were analyzed using frequency counts; thematic analysis was also conducted to identify key concepts. Survey responses were anonymous; interviews were not recorded. Most subjects offered information that would be considered identifying to others in the field. As such, all data was de-identified and aggregated, removing references to specific events or locations.

### 3.4. Limitations

There are limitations to the external validity and generalizability of case study results [5], particularly with regard to the small sample size of survey/interview respondents. Any inferences drawn from differences between the groups of respondents (outbreaks detected quickly, slowly, and other) should be considered with caution, given small sample sizes. Indeed, these case studies may not be generalizable to all zoonotic disease outbreaks: each outbreak is unique. Additionally, due to confidentiality concerns, detailed information cannot be provided on specific aspects of each outbreak. Finally, this analysis may not be generalizable to other systems of governance—particularly those that are not federalized.

## 4. Results and Discussion

### 4.1. Literature Review of Cases

In terms of the two slow cases, the first outbreak affected only humans; both humans and domestic pets (such as hamsters and mice) were affected during the second outbreak. The literature highlighted that both were foodborne infectious that were ultimately identified through PulseNet, a system operated by the Centers for Disease Control and Prevention which uses DNA information to detect outbreaks [29]. The pulsed-field gel electrophoresis (PFGE) subtyping allowed astute epidemiologists to identify an increase in specific strains of Salmonella in PulseNet. However, there was a significant lag between the first symptoms and PulseNet analysis to detect the outbreak. In large part, this is due to the fact that cases—initially not thought to be related—were later linked to the outbreak through retrospective analysis of PFGE patterns in PulseNet.

Both slow cases involved many states—as many as 21 in one outbreak [30]. States were geographically dispersed, such as cases in both Minnesota and South Carolina [31]. The literature indicates the detection of the outbreak was impeded by the extent to which the cases were “dispersed temporally and geographically” [32]. Additionally, Salmonellosis typically has low fatality rates in humans, though many rodents died in one of the two cases [31]. This may have contributed to the delay in detection. Ultimately, without the PFGE suptyping that was aggregated in PulseNet for further analysis, it is unlikely that either of these outbreaks would have been characterized or successfully investigated to a source.

In terms of the fast cases, the rapid onset of severe rabies symptoms probably aided in detection; additionally, the astute clinician rapidly identified confounding risk-factors for the disease [33]. Even though rabies is not frequently observed in the United States, unlike many tropical diseases, clinicians are frequently aware of clinical signs that may indicate rabies infection. The second fast case—like the second slow case—affected both humans and animals, in this case, livestock. This outbreak affected areas historically unaffected by anthrax, and resulted in a significant increase in anthrax cases in cattle [34]. Given the large outbreak and the patient’s direct contact with dead cattle, the clinician rapidly gave a presumptive diagnosis of anthrax which was later confirmed in the laboratory. This was the first case of cutaneous anthrax in the United States for eight years [34], and preceded the 2001 attacks with *Bacillus anthracis*.

The literature provides important information which aids in describing the detection of these four cases. It is clear that consistent and multi-state use and access to PulseNet is critical for detection, and that the frontline providers are often key in making rapid detections. However, the literature does not provide much insight into whether federalism, bureaucratic behavior, or institutional design that may have impacted detection. As such, we turn now to the interview and survey which specifically explored these intersections.

### 4.2. Interview and Survey Results

Thirty individuals, with a direct role in detecting zoonotic disease outbreaks, were asked to participate in the research, with a response rate of 60 percent (*n =* 18). Of these respondents, eight were from the fastest cases to detection (44%), six from the slowest cases (33%), and four from other outbreaks (22%). Additionally, three individuals were not willing to participate in the research due to confidentiality concerns, or because they did not feel as if they could recall the events correctly. This response rate is typical of survey instruments [35]. Of these respondents, 50 percent completed the survey and 50 percent were interviewed by phone. While there is no doubt this is a small sample size, there are a limited number of individuals that have been directly involved in the detection of a zoonotic disease outbreak, and this sample represents a key number of those individuals.

Table 1 provides a breakdown of respondents by level of government and sector. Table 2 indicates role of respondent. Further disaggregation of these results is not possible due to confidentiality concerns.

**Table 1:** Response Information for Survey/Interview by Level of Government and Sector.

Sector/Level	Local	State	Federal	Total
Human Health	2	7	2	11
Animal Health	0	5	2	7
Total	2	12	4	18

**Table 2:** Role of Respondent.

Role	Total
Laboratory	3
Official/ Practitioner	15
Total	18

#### 4.2.1. Impediments to and Facilitators of Detection

Respondents were specifically asked to identify impediments and facilitators to detecting zoonotic disease outbreaks, in order to ascertain whether issues of governance arose naturally. They were requested to answer with a specific outbreak in mind—either one of the slow or fast cases identified previously, or another outbreak as identified in their response. Table 3 lists the top three impediments identified; Table 4 lists the top three facilitators for each group of respondents (outbreaks detected slowly, outbreaks detected rapidly, and other outbreaks).

**Table 3:** Top Three Impediments for Zoonotic Disease Outbreak Detection and Reporting.

Impediment	Response Percentage (*n =* 18)	For Outbreaks Detected Rapidly (*n =* 8)	For Outbreaks Detected Slowly (*n =* 6)	For Other Outbreaks (*n =* 4)
Knowledge/obligation to report	44%	38%	17%	100%
Diagnostics	33%	50%	17%	25%
(tie) Astute, Aware, Educated Providers	22%	25%	33%	0%
(tie) Funding	22%	13%	33%	25%

**Table 4:** Top Three Facilitators for Zoonotic Disease Outbreak Detection and Reporting.

Facilitator	Response Percentage (*n =* 18)	For Outbreaks Detected Rapidly (*n =* 8)	For Outbreaks Detected Slowly (*n =* 6)	For Other Outbreaks (*n =* 4)
Diagnostics	44%	63%	50%	0%
Communication and Collaboration between Agencies and Practitioners/Laboratories	39%	50%	50%	0%
Astute, Aware, and Educated Providers	33%	25%	33%	50%

For impediments, respondents specifically mentioned the lack of knowledge regarding both federal and state reporting requirements and how to report diseases. Specific impediments included the lack of awareness amongst providers and practitioners about reporting requirements and law, resource or time burdens of reporting, and the pressure from clients (both on the veterinary and human side) not to report. For diagnostics, respondents primarily mentioned the need for and lack of rapid, cost-effective, sensitive, and specific diagnostic tests, particularly for diseases less commonly observed in the United States. A lack of electronic reporting was also noted as a gap. Respondents also cited the gap of awareness, education, and training amongst providers and practitioners regarding zoonotic disease threats, and the understaffed and under-resourced nature of many local and state departments which work to detect zoonotic disease outbreaks.

It is possible to note some differences between the groups of respondents. Those involved in “fast” outbreaks more frequently cited that a lack of knowledge regarding reporting requirements and diagnostics impeded rapid outbreak detection more than those who responded from “slow” outbreaks. Those involved in “slow” outbreaks spoke more often about a lack of astute and trained providers and funding issues. Given the criticality of diagnostics and electronic reporting in detecting the “slow” outbreaks, it is not surprising that this group of respondents did not cite this as a significant impediment to detection. Interestingly, all of the respondents involved in the “other” disease outbreaks cited that a lack of knowledge about disease reporting requirements impeded outbreak detection.

Diagnostics emerged at the top of the list for facilitators. Rapid, sensitive, and specific diagnostic testing was cited by both groups of respondents (“slow” and “fast” cases) as a key factor that facilitated outbreak detection and reporting. Interestingly, those responding from the “other” group did not mention diagnostics. Respondents from both the rapidly and slowly detected outbreaks frequently mentioned that communication and cross-agency cooperation, as well as collaboration between practitioners and laboratories facilitated detection. Just “picking up the phone to discuss” was often mentioned as a way to facilitate detection. Finally, astute and trained providers can be critically important to rapidly detecting and reporting outbreaks with the appropriate training and keen knowledge particularly on asking the right questions to capture suspected zoonotic disease exposures. However, it is interesting that the respondents for the rapidly detected outbreaks did not mention this more frequently, given the importance of this factor in the detection and reporting of those rapidly detected outbreaks.

#### 4.2.2. Collaboration between Sectors

Next, respondents were specifically asked about the collaboration and coordination between animal health and human health agencies/entities in detecting the specific outbreak. This question resulted in some of the most diverse responses characterizing the relationship, from “miserable” to “contentious” to “we worked great together” and “there were and are no issues”. Interestingly, nearly all respondents (89%) volunteered that they felt as if this issue varied dramatically by state.

The majority of respondents from both the human health side (63%) and animal health side (57%) reported that their working relationship between the two sectors was positive. Many provided concrete observations of bilateral press releases, publications, and outreach to ensure a consistent public message. Of those reporting a problem (*n =* 6), most were linked to outbreaks that were detected slowly (67%). Interestingly, the individuals noting problems between the two sectors focused on response to zoonotic disease incidents rather than detection to the incidents. Additionally, respondents stated problems with not working towards a common goal successfully and problems or barriers to effective information sharing.

#### 4.2.3. Federalism

When explicitly asked about the role of federalism in zoonotic disease detection and reporting, only a single respondent suggested that federalism played a role. Interestingly, this respondent also noted that federal reporting requirements for specific diseases did impact detection and reporting. However, an additional three respondents noted that federalism was not important in detection or reporting, but was critically important in response; collaboration between state and federal agencies during the response phase was problematic. Though respondents did not recognize federalism as playing a role when questioned explicitly, four individuals did offer that individuals at the local level are not provided enough deference by state and federal officials, who must conduct their own investigations into the disease outbreak. Responses were equally distributed across the fast, slow, and other cases.

#### 4.2.4. Institutional Design and Bureaucratic Behavior

In comparison to federalism, many more respondents (50 percent or *n =* 9) suggested that institutional design and bureaucratic behavior play a role in zoonotic disease detection and reporting. These nine respondents included respondents from the slow (*n =* 3), fast (*n =* 4), and other (*n =* 2) cases; or 50% of the respondents from each of these groups. Of these nine respondents, 44 percent (*n =* 4) noted that they felt that institutional design and bureaucracy was actually a positive factor; three others suggested it was negative, and two thought it was both positive and negative. In all, six of the nine respondents stated that institutional design and bureaucratic behavior could or did play a positive role; in their remarks, respondents cited their own state system as a “model” of a bureaucracy that was effective for practice. Regarding the potential negative influence of bureaucratic behavior on zoonotic disease detection, comments specifically focused on behavior and lack of coordination or communication amongst executive agencies, resulting in “siloes” and “misguided budget priorities”.

#### 4.2.5. Other Factors

Finally, respondents were asked if there were other factors, not yet discussed, that could improve U.S. zoonotic disease detection and reporting, based on their experience in the field. These responses were not broken out between the groups as respondents replied “in general” rather than with reference to a specific outbreak. Interestingly, 10 of the 18 respondents specifically cited interpersonal relationships: knowing the person on the other end of the phone, in a different sector, that they could speak to both officially and informally. Table 5 lists the other responses to this question.

**Table 5:** Top Three Improvements Suggested for Zoonotic Disease Detection and Reporting.

Improvement	Response Percentage (*n =* 18)
Interpersonal Relationships/Communication	67%
Education (Practitioner and Public)	22%
Resources	16%

Resources and education/training (which requires resources) are commonly discussed factors for improving zoonotic disease detection and reporting for example, [36]. While outside the scope of this research, it is unclear to what extent the outcome—faster disease detection, reporting, and ultimately response—is linked back to specific inputs. Given an era of fiscal uncertainty and tight budgets—particularly at the state and local level—it is important that resources be used as effectively as possible. However, while the argument can most definitely be made that zoonotic disease detection requires more resources [7,8], determining the most appropriate allocation of these resources remains difficult.

### 4.3. Discussion

Theoretical literature suggests that federalism, institutional design, and bureaucratic behavior could play a significant—and by most accounts—detrimental role in zoonotic disease detection. Indeed, there are pieces of empirical evidence that suggest the same. However, there is also information which suggests that these factors could play a role in facilitating rapid zoonotic disease detection. This section connects the gathered empirical evidence on outbreak detection—in particular, the responses to the interview/survey instrument—with the theoretical literature on federalism, bureaucratic behavior, and institutional design.

The factors listed in Table 3 and Table 4 are well in line with those involved in zoonotic disease detection and reporting. Indeed, the literature review on these outbreaks also indicated the importance of diagnostics (including electronic reporting, as indicated in the outbreaks that were detected slowly) and astuteness of providers in disease detection. However, the more important question is: are these factors influenced by federalism, bureaucratic behavior, or institutional design? Certainly, three of the factors can be connected back to issues of governance.

First, and perhaps most importantly, is the knowledge or willingness to report notifiable diseases (Table 3). This factor was listed by respondents as the number one impediment to rapid detection. As a direct result of a federalized system where powers are reserved to the states, each state has varying requirements for reporting (e.g., how fast and who has to report); reporting of many notifiable diseases is often voluntary from the state level to the federal level. This regulatory framework may be detrimental to rapid zoonotic disease detection, particularly in multi-state outbreaks where these inconsistencies across states can easily delay recognition and/or response. It has been suggested that a more consistent regulatory approach and additional state to federal reporting requirements (and penalties for failure to report) would “encourage” and facilitate detection and reporting.

Second, respondents cited communication and collaboration across agencies and roles as an important facilitator in detecting and reporting past outbreaks. Interestingly, many theories of bureaucratic behavior indicate that executive agencies may choose to compete or compartmentalize information rather than cooperate. Indeed, this was reflected in the responses to the question regarding collaboration between the sectors (animal and human): one respondent suggested that absolutely no interaction occurred between the two sectors (animal health and human health) in the zoonotic disease outbreak which hampered detection and response; a concerning matter which has also been identified in past research [37]. However, respondents also suggested that effective cooperation between agencies occurs regularly in certain states, leading to positive outcomes for human and animal health.

Third, funding was reported as an impediment by respondents as well as a top three need to improve zoonotic disease detection and reporting. Resources were also mentioned in the outbreak literature review and predicted by theories of institutional design and behavior: some agencies have significantly more resources and/or influence, based on their design and behavior interacting with stakeholders. Moreover, in a federal system, each state operates with different capabilities and funding allocations based on the interests of the state. As noted by respondents and literature on zoonotic disease, deficient levels of funding can result in a lack of personnel, old IT systems, insufficient diagnostic equipment, or an absence of zoonotic disease education in either the animal or human health sectors—all of which could potentially delay zoonotic disease detection.

Despite the fact that implicitly some of the respondents’ remarks regarding factors that facilitate or impede zoonotic disease detection can be identified as a consequence of federalism, respondents did not explicitly state that federalism played an important role in facilitating or impeding zoonotic disease detection. This is particularly interesting given the number of comments about state-specific success in zoonotic disease detection—a result of a federal system where states are independent actors with delegated and reserved powers. Some states may develop highly effective ways to facilitate interaction and collaboration facilitating disease detection. However, these comments are consistent with other research, where states have emerged as innovators [38] in both disease detection and reporting practices. Federalism—with the ability for states to create their own infrastructure, processes, regulations, and organizations—can facilitate detection. Electronic reporting, formal pathways between animal health and human health agencies, close relationships with producers and farm veterinarians, as well as informal training and workshops were all mentioned as specific mechanisms to facilitate zoonotic disease detection within a state.

Respondents did both implicitly suggest and explicitly state that bureaucratic behavior and institutional design affected the detection and reporting of the zoonotic disease outbreak: half of the respondents believed there was an impact. However, six of these nine respondents suggested that their experiences with bureaucratic behavior were not negative. This empirical evidence reinforces results found in an examination of the 1999 West Nile Virus outbreak in New York State [39], where initially the author notes questions of jurisdiction and authority (both between levels of government and across sectors of government) but finds that intergovernmental operations were also flexible, adapting and acting effectively across jurisdictions and sectors. While this is a divergence from what is commonly suggested in the theoretical literature—that executive agencies tend to compartmentalize information and fail to coordinate effectively—it is important to note that there were also respondents who clearly indicated that coordination problems and information siloes remain. It appears that certain states have better communication and collaboration processes in place across executive agencies, and as such, the behavior of bureaucracies and institutions and experience of officials and practitioners varies by state. Flexibility and adaptability of regulatory processes and ease of communication in matters of disease detection and reporting appears critical to rapid detection.

In sum, it is clear that federalism may impact the timeliness of zoonotic disease detection and reporting: while respondents did not explicitly cite federalism, implicitly they relayed multiple remarks demonstrating that regulatory differences across states and between states and the federal government is problematic, as also indicated in the outbreak and theoretical literature review. However, the ability of states to form and shape their own institutions and processes can also facilitate rapid detection through efficient collaboration. A federal system of governance therefore has multiple consequences for zoonotic disease detection with opposite effects. The evidence collected here on the impact of bureaucratic behavior and institutional design also remains mixed, though ultimately the impact of these factors appears to vary by state and outbreak circumstances. It seems likely that effective behavior and well-designed institutions and processes can facilitate detection, while compartmentalization of information, funding issues, and poor cooperation between executive agencies (between the animal and human sectors) can all impede detection of zoonotic disease outbreaks.

## 5. Conclusions

While researchers and policy analysts yearn for clear conclusions, the extent to which the U.S. system of governance impacts zoonotic disease detection and reporting deserves significantly more attention. Characteristics of the U.S. system of governance do matter in the success or failure of One Health approaches to key problems in policy and practice. This is the first attempt in the literature to describe the area of intersection between public policy and administration and One Health.

While empirical evidence suggests compartmentalization of knowledge within the animal health and human health sectors, as well as within different levels of government, innovative local and state systems can facilitate efficiency and flexibility. Bureaucracies can be flexible and adaptable, while also being prone to funding issues and overlapping regulations. After action reviews and reports of lessons learned for disease outbreaks must not only capture the technical side of outbreak detection—including the effectiveness of surveillance systems, epidemiological investigations, and disease control—but also the public policy and administration aspect to fully comprehend the facilitators and impediments to rapid detection and reporting. Moreover, other factors matter beyond those of governance, including technical advances and interpersonal relationships.

Further research on the topic is crucial, particularly from those federal, state, and local personnel responding to zoonotic disease outbreaks in the United States. This research can help to inform improvements in zoonotic disease detection, reporting, and response at the local, state, and federal levels by raising awareness of these complex issues. Systematic analysis of this intersection is critical to better understanding how to successfully execute One Health activities in a complex U.S. federal system of governance.
